# Diachronic semantic change in language is constrained by how people use and learn language

**DOI:** 10.3758/s13421-022-01331-0

**Published:** 2022-06-29

**Authors:** Ying Li, Cynthia S. Q. Siew

**Affiliations:** 1 CAS Key Laboratory of Behavioral Science, Institute of Psychology, Chinese Academy of Sciences, 16, Lincui Road, 100101 Beijing, China; 2 Present Address: Center for Adaptive Rationality, Max Planck Institute for Human Development, Lentzeallee 94, 14195 Berlin, Germany; 3 Department of Psychology, National University of Singapore, 9 Arts Link, 117570 Singapore, Singapore

**Keywords:** Language evolution, Semantic change, Age of acquisition, Semantic decision, Lexical decision

## Abstract

While it has long been understood that the human mind evolved to learn language, recent studies have begun to ask the inverted question: How has language evolved under the cognitive constraints of its users and become more learnable over time? In this paper, we explored how the semantic change of English words is shaped by the way humans acquire and process language. In Study 1, we quantified the extent of semantic change over the past 200 years and found that meaning change is more likely for words that are acquired later in life and are more difficult to process. We argue that it is human cognition that constrains the semantic evolution of words, rather than the other way around, because historical meanings of words were not easily accessible to people living today, and therefore could not have directly influenced how they learn and process language. In Study 2, we went further to show that semantic change, while bringing the benefit of meeting communicative needs, is cognitively costly for those who were born early enough to experience the change: Semantic change between 1970 and 2000 hindered processing speeds among middle-aged adults (ages 45–55) but not in younger adults (ages <25) in a semantic decision task. This hampering effect may have, in turn, curbed the rate of semantic change so that language does not change too fast for the human mind to catch up. Taken together, our research demonstrates that semantic change is shaped by processing and acquisition patterns across generations of language users.

**Supplementary Information:**

The online version contains supplementary material available at 10.3758/s13421-022-01331-0.

## Introduction

Languages face the challenge of expressing an infinite range of ideas using a finite set of words (Chomsky, 1957). While one way to meet this challenge is to create new words to express novel meanings, a more common strategy is to assign new meanings to existing words (Ramiro et al., 2018). Previous literature has shown that the meanings of words change in predictable ways (Ullmann, 1962) and various accounts of such regularities in semantic change have been proposed, such as grammaticalization, subjectification, erosion, and metaphorization (Lakoff, 2008; Traugott & Dasher, 2001). In addition, there are case studies that documented how and when individual words changed their meaning (e.g., Lehrer, 1985). For instance, the original meaning of the word *broadcast* in agriculture was to cast or sow seeds widely, but with the advent of communication technologies (such as the radio), *broadcast* (since the early 20th century) refers to the spreading or transmitting of a message.

What factors make some words change their meanings to a greater extent than others? Our approach to this question follows a Darwinian view that considers language as a complex adaptive system that evolves under the selection pressure of human cognitive constraints over generations of language users (Beckner et al., 2009; Christiansen & Chater, 2008; Darwin, 1871). One important cognitive constraint that may have shaped language evolution is general learning and processing biases derived from cognitive limitations. One of the advantages of such a view of language evolution is that apart from explaining how human language emerges, it also provides a theoretical foundation to explore hypotheses of how human languages *change* after their emergence, which is the scope of the current study.

Evidence supporting the relation between how language is acquired and processed and how language evolved has been accumulating, involving laboratory-based experiments of artificial languages (Kirby et al., 2008), computational simulations (Kirby, 2001; Monaghan et al., 2011; Steels, 2011), and more recently, analysis of natural language corpora (Hills & Adelman, 2015; Monaghan, 2014). Moreover, it is worth pointing out that cognitive constraints do not have to be very strong to shape language evolution. Even weak individual-level biases in language behaviors can be scaled up to population-level linguistic phenomena via cultural transmission (e.g., Scott-Phillips & Kirby, 2010) or social structure (e.g., Linguistic niche hypothesis; Lupyan & Dale, 2010).

However, to date, most studies demonstrating the relation of language evolution to language acquisition and processing have focused on evolution of the lexical form (e.g., Monaghan, 2014; Pagel et al., 2007). Less evidence exists for how the evolution of *semantics,* or the meanings of words, is related to language acquisition and processing. Fortunately, the availability of large-scale diachronic language corpora, along with computational tools for quantifying diachronic change in word embeddings over time (e.g., Dubossarsky et al., 2016; Hamilton et al., 2016; Li et al., 2019) have enabled us to address this particular gap in the literature.

Semantic change could be viewed as the result of competing selective forces that arise from learners (Christiansen & Chater, 2008), as well as listeners and speakers (Zipf, 2016) who are using language within a fast-changing social and technological landscape (Jones, 2016). The rapidly changing information landscape (Eppler & Mengis, 2004; Varian & Lyman, 2000), particularly with the advent of new and emerging technologies and increased cultural contact and interaction, is likely to have placed increasing demands on language users to express new meanings. Even though inventing new lexical forms for every new meaning is probably favorable for the listener (because each lexical form would unambiguously signal a unique meaning), such an approach will make the size of the lexicon excessively large and consequently difficult for all language users to learn, produce, and process. Therefore, new meanings are often expressed by existing lexical forms (Ramiro et al., 2018).

This leads to the question of which words are more likely to host new meanings? Previous studies have addressed this question by identifying linguistic features (e.g., word frequency, syntactic class, polysemy, prototypicality) that make a word more susceptible to semantic change than others (Dubossarsky et al., 2016; Hamilton et al., 2016; Pagel et al., 2013). Another approach to the same question is to consider the cognitive pressures that generations of language users impose on language evolution. Early acquisition and ease of processing may directly act as preservers against semantic change. Psycholinguistic research has shown that early-acquired words are used more frequently (Balota et al., 2007; Pexman et al., 2017), retrieved more quickly and accurately (Juhasz, 2005), and tend to be more resistant to the onset of aging (Hodgson & Ellis, 1998) and to acquired cognitive impairment (Bradley et al., 2006). Hence, early-acquired words should be less vulnerable to change due to the cognitive prioritization that early-acquired words are afforded.

Other than early acquisition, we speculate that ease of processing also “protects” words from semantic change. It takes longer for people to process (that is, derive meaning from words) for words with many distinct meanings (homonyms) than words with few or related meanings in semantic tasks (Hino et al., 2006; Rodd et al., 2002); the latter set of words may be easier to process because of the stronger association between the lexical form and meaning such that the lexical form is a reliable signal of its overall meaning. Such words would be less suitable for hosting new meanings since the cost to re-associate such lexical forms with a new meaning would be high, ultimately decreasing learnability of new form-meaning mappings. On the other hand, words that are difficult to process are likely to have less well-established form-meaning associations (i.e., the word form is a weaker signal of meaning), reducing the cost of updating the meanings of these words. Moreover, reassociating with new meanings provides opportunities for the lexical form to evolve toward the direction of evolutionary success, particularly if the new meaning becomes frequently used in the current environment.

## Overview of current study

**Fig. 1:**
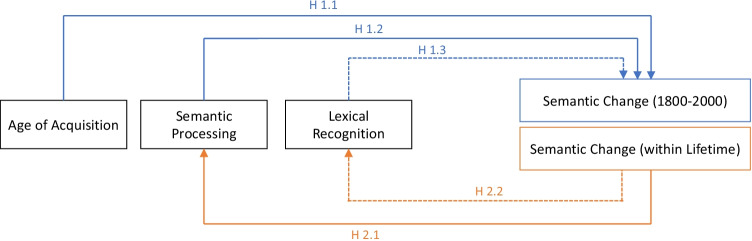
Graphical representation of causal relationships examined in Study 1 (in blue) and in Study 2 (in orange). Dotted arrows represent causal relationships that were hypothesized to *not* exist. (Color figure online)

The present study first explores whether early acquisition and ease of processing lead to higher rates of semantic change. In Study 1, the age at which words are acquired and semantic processing speed were tested as predictors of the rate of semantic change over the past 2 centuries. We hypothesize that words that are acquired later in life and are more difficult to process changed their semantics to a greater extent (H1.1, H1.2; Fig. 1). In Study 1, note that we did not examine the inverse causal relation where semantic change since 1800 led to difficulty in learning and processing in modern times. This is because the historical meanings, especially those existed long *before* people today were born, were not readily accessible to people living today, and therefore should have little direct influence on how people today learn and process language^1^ (see Appendix section 1).

After testing hypotheses of the cognitive factors that drive semantic change, in the second study we turned to a related question: the cognitive cost of semantic change. We reasoned that there should be a “sweet spot” in the rate of semantic change: fast enough to meet the ever-changing demand for expressing new meanings, but not too fast for the human mind to catch up. After all, a highly unstable language whereby the meanings of all words are changing constantly is unlikely to be learnable by humans, and unlikely to result in effective communication. Therefore, we expect that semantic change of words, if and only if it has occurred during one’s lifetime, would hamper semantic processing. Leveraging on existing mega study databases and complemented with new data from psycholinguistic experiments, we examined how the rate of semantic change between 1970 and 2000, which is only personally experienced by middle-aged adults (ages 45–55) but not by younger adults (ages 18–25), affected the semantic processing of older and younger participants. Based on previous research showing that slower processing speeds among middle-aged adults could be attributed to interference and information accumulation in long-term memory (Ramscar et al., 2017; Qiu & Johns, 2020), we reasoned that exposure to inconsistent semantic meanings of a word over one’s lifespan may inadvertently activate deprecated meanings and therefore slow down processing speeds due to this interference effect. We hypothesize that higher rate of semantic change has a stronger effect in slowing down the speed of semantic processing for middle-aged adults than younger adults. (H2.1; Fig. 1)

Lastly, we investigated whether lexical recognition performance in a visual lexical decision task (tell whether a string of letters is a word or nonword) is related to semantic change. Since such tasks tend to show strong effects of familiarity to the word form (Balota & Chumbley, 1984) rather than measuring specific aspects of a word’s semantic representation (but see Pexman et al., 2002, for an example showing semantic effects in word recognition), we expected that rate of semantic change is not related to performance in the lexical decision task in both studies (H1.3, H2.2; Fig. 1)

Our specific hypotheses are as follows (see Fig. 1):

Study 1:

- H1.1 Words acquired later in life experience greater semantic change than words acquired earlier in life.
- H1.2. Words that are processed slower in the semantic decision task experience greater semantic change than words that are more quickly processed.
- H1.3. Performance on a visual lexical decision task is not related to semantic change of words.

Study 2:

- H2.1. Semantic change of words, when personally experienced within one’s life span, slows down semantic processing. Specifically, semantic change of words slows semantic processing of middle-aged adults more than for younger adults.
- H2.2. Performance on a visual lexical decision task is not related to semantic change of words (a second test of H1.3 using a different dataset).

## Methods and materials

### Quantifying rate of semantic change

Taking a Firthian approach (Firth, 1957), we assumed that the meaning of a word can be reliably inferred from the linguistic contexts in which the word has been used in. Therefore, the semantic shift of a word between two time points in history can be captured by comparing the extent to which its context has changed. Therefore, our approach captures (but does not distinguish) the shift in two kinds of meanings: denotation (meaning that can be looked up in dictionary) and connotation (associations evoked by words in the mind of readers). For example, although the denotative meaning of *woman* (adult female human being) remained the same over the past 200 years, our approach to word meaning would suggest that the meaning of *women* has changed because its connotation, reflecting the rising socioeconomic status of women, has become increasingly associated with characteristics traditionally perceived to only belong to men (Garg et al., 2018).

### Historical corpora

We used the English Google Ngram Corpus (Michel et al., 2011) to extract contextual information of words for each year from 1800 to 2000. To ensure that our findings are not an artifact of a specific corpus, we validate our findings on the Corpus of Historical American English (COHA; Davies, 2012).

The Google Ngram Corpus represents around 6% of all books published over the past several hundred years, which contain approximately 155 billion words (Michel et al., 2011). On the one hand, it has proved fruitful in capturing cultural shifts such as evolution of grammar, adoption of technology (Michel et al., 2011), and national well-being (Hills et al., 2019); on the other hand, it has been criticized to contain corpus artifacts due to its shifting sampling paradigm (e.g., a surging proportion of academic articles as observed in Pechenick et al., 2015). In contrast, the COHA corpus is much smaller in size (400 million words from the 1810s to the 2000s). The COHA is carefully selected to be genre-balanced over each decade, which has its advantages and disadvantages. On the one hand, it alleviates concerns that insights gained from the corpus are driven by changing compositions of genres. On the other hand, it may fail to reflect the reality that public preferences for genres do change over history. Although it is difficult to argue whether COHA is a better corpus for analyzing language change than the Google Ngram Corpus or vice versa, consistency in the findings from both corpora would lend convergent validity to the results.

### Embedding algorithm

The meaning of words can be quantified through the use of distributional semantics, in which words are represented by numeric vectors (often referred to as *word embeddings*) in accordance with their co-occurrence relationships (Bullinaria & Levy, 2007). Hamilton et al. (2016) trained word embeddings from both Google Ngram Corpus and COHA using three algorithms: Positive Pointwise Mutual Information (PPMI), Singular Value Decomposition (SVD) and word2vec. They evaluated the three algorithms in terms of their performance in detecting a set of independently attested semantic shifts. They found that SVD performs consistently well on both Google Ngram Corpus and COHA while PPMI and word2vec performed poorly on at least one of the two corpora (Appendix Table 2.1). Therefore, in this study, we used SVD to quantify rate of semantic change.^2^

One potential weakness of SVD is that large semantic change may be an artifact of change in frequency: Because the contexts in which low frequency words appear in may be less representative, estimates of their semantics could be unreliable. However, when analyzing Hamilton et al.’s (2016) word embeddings trained using SVD, we found that the correlation between the rate of semantic change and change of frequency is actually quite small (*r* = −.05, *p* < .001 for Google Ngram Corpus; *r* = .31, *p* < .001 for COHA; for full details, see Appendix Table 2.3).

We obtained diachronic word embeddings trained on the Google Ngram Corpus from Li et al. (2019), and diachronic word embeddings trained on the COHA from Hamilton et al. (2016). Both word embeddings were trained using SVD, which were constructed based on the following steps. First, a co-occurrence matrix was constructed to record the number of times any two words co-occurred within fixed-size sliding windows of text. Second, vectors containing the number of times a given word co-occurred with all other words were directly obtained from the co-occurrence matrix described above. Third, they computed the PPMI for each pair of words and then constructed a PPMI matrix with entries given by

1 $$\mathrm{PPMI}\left({v}_i,{v}_j\right)=\max \left(0,\log \left(\frac{P\left({v}_i,{v}_j\right)}{P\left({v}_i\right)\times P\left({v}_j\ \right)}\right)\right),$$

where *v*_*i*_*, v*_*j*_ represents a pair of words from the corpus. *p(v*_*i*_*,v*_*j*_*)* corresponds to the empirical probabilities of word co-occurrences within a fixed-size sliding window of original text. As compared to co-occurrence counts, PPMI penalizes high-frequency words (i.e., *of, the, and*) that were used in a wide range of contexts and favors word pairs that frequently appeared together but not with others (i.e., *Hong* and *Kong*). Forcing PPMI values to be above zero ensures that they remain finite, and this has been shown to improve results (Bullinaria & Levy, 2007). Finally, dimensionality of word embeddings was reduced to 300 using singular value decomposition (SVD). This dimensionality reduction acts as a form of regularization and allows us to compare word similarities by computing the cosine similarity of word embeddings. This approach has been effectively demonstrated in several studies (Hamilton et al., 2016; Li et al., 2019; Sagi et al., 2011; Xu & Kemp, 2015).

With diachronic word embeddings, the semantic stability (i.e., the inverse of the rate of semantic change) of a given word can be quantified as

2 $${SemanticStability}^{T1,T2}\left({w}_i\right)={\mathit{\cos}}_{dist}\left({w}_i^{(T1)},{w}_i^{(T2)}\right),$$

where ${w}_i^{(T)}$ refers to the word embedding of word *w*_*i*_ in year *T*. The historical embedding is aligned to its modern embedding using orthogonal Procrustes (Hamilton et al., 2016; Schönemann, 1966). Semantic similarity ranges from 0 to 1. For example, the semantic similarity of *happy* between year 1800 and 2000 is 0.73, much higher as compared to words that had undergone greater semantic change, such as *gay* (0.36), and *car* (0.41).

**Fig. 2:**
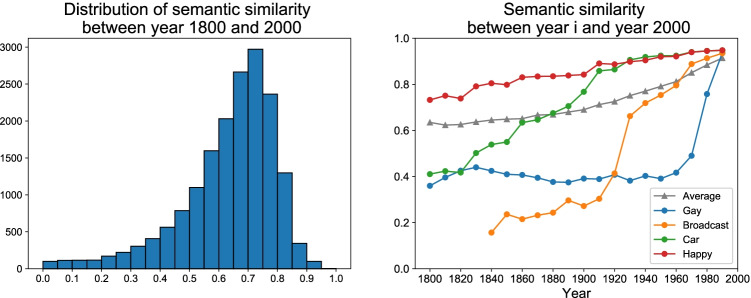
Both figures were produced based on the Google Ngram Corpus. Left: Distribution of semantic similarities between 1800 and 2000. Right: Semantic stability of selected words. Each line represents the semantic similarity between the historical meaning (across years 1800–1990) and the contemporary meaning (year 2000) of the corresponding word. The grey line represents the average semantic similarity across all words in the Macroscope database (Li et al., 2019). (Color figure online)

Figure 2 (left) shows the distribution of semantic similarity between 1800 and 2000 for 50,000 English words trained on the Google Ngram Corpus. The negatively skewed distribution suggests that the majority of words were used in similar contexts at both time points. Figure 2 (right) shows the semantic stability of a few words as examples. Each line represents the semantic similarity between its historical meaning (across years 1800–1990) and its contemporary meaning (year 2000) of the corresponding word. The average semantic stability of the entire database is plotted in grey as a benchmark. Figure 2 (right) suggests that the word *happy* is relatively stable in its semantics over the past 2 centuries. In contrast, *gay*, *car*, and *broadcast*^3^ all changed their meanings drastically. The turning points suggest that the semantics of *gay* changed (from “joy” to “homosexuality”) roughly in the 1950s, whereas the semantic change of *broadcast* (from “spread of seed” to “spread of information”) took place earlier in the 1920s, and semantics of *car* (from “wheeled horse-drawn vehicle” to “automobile”) changed gradually over the entire 19th century.

## Study 1

### Method

In Study 1, we tested hypotheses H1.1, H1.2 and H1.3. The materials we used in both studies are summarized in Table 1. To test H1.1 (Words acquired earlier in life are more likely to be semantically stable), we used Age of Acquisition ratings (AoA) collected by Kuperman et al. (2012). In their study, participants were asked to report the age (in years) at which they thought they had learned the word.^4^ This self-report measure of age of acquisition has been validated on a more naturalistic measure based on vocabulary assessment among pupils (Pearson *r* = .76; Brysbaert & Biemiller, 2017). To test H1.2 (words processed faster in a semantic decision task are more likely to be semantically stable), we used data from the Calgary Semantic Decision Project (Pexman et al., 2017). In the semantic decision task, participants had to decide, as quickly as possible, whether a word was abstract or concrete. A total of 321 participants provided abstract-concrete decisions to 10,000 English words. To test H1.3 (lexical recognition speed is less strongly related to semantic change of words as compared to semantic processing speed), we used visual lexical decision data from the English Lexicon Project (Balota et al., 2007). In contrast to the semantic decision task that requires participants to retrieve the semantic meaning of words, the lexical decision task requires participants to decide, as quickly as possible, whether a string of letters formed a word or a nonword. This dataset contained mean reaction time and accuracy rates for 40,481 words and 40,481 nonwords collected from 444 participants.

**Table 1:** Information about the datasets used to test our hypotheses

	Data	Hypothesis	Participants	Age	Number of words
Study 1	Age of Acquisition ratings (Kuperman et al., 2012)	H1.1	1,729 MTurk workers	15–82	30,121 (8,133/2,845)
	The Calgary Semantic Decision Project (Pexman et al., 2017)	H1.2	321 college students	Mean: 21.2 *SD*: 5.8	10,000 (4,847/2,064)
	The English Lexicon Project (Balota et al., 2007)	H1.3	816 college students	Mean: 22.8 *SD*: 5.8	40,481 (2,786/887)
Study 2	Newly collected data on semantic decision task	H2.1	237 recruited from Prolific	Two age groups: Group 1: 18–25 Group 2: 45–55	180
	The English Crowdsourcing Project (Mandera et al., 2019)	H2.2	584,284 online volunteers	Mean: 35.5 *SD*: 14.7	62,000

Both the number of words in the dataset and the number of words used in the analysis (in parentheses, Google Ngram Corpus/COHA) are reported. Only the words that had values for all linguistic properties in the regression model were analyzed.

For each corpus, we investigated how semantic change was related to AoA, semantic processing, and lexical recognition in three regression models where semantic stability was regressed on AoA, response time of either lexical decision task or semantic decision task, and other lexical-semantic variables such as log frequency, length, emotionality, arousal, and concreteness that are known to correlate with lexical retrieval (see Table 2). Response time was computed for each word by averaging response times across all participants. All predictors were scaled and mean-centered.

**Table 2:** Summary of regression models analyzed in Study 1

	Google Ngram Corpus						COHA					
	Predicting Semantic Stability Between 1800 and 2000						Predicting Semantic Stability Between 1820 and 2000					
*Predictors*	*Estimates*	*p*	*Estimates*	*p*	*Estimates*	*p*	*Estimates*	*p*	*Estimates*	*p*	*Estimates*	*p*
(Intercept)	0.00	1.000	0.07	**<0.001**	0.09	**<0.001**	-0.93	**<0.001**	-0.74	**<0.001**	-1.01	**<0.001**
Log Frequency (year 1800/1820)	0.73	**<0.001**	0.73	**<0.001**	0.76	**<0.001**	0.63	**<0.001**	0.48	**<0.001**	0.65	**<0.001**
Log Frequency (year 2000)	-0.67	**<0.001**	-0.64	**<0.001**	-0.74	**<0.001**	0.18	**<0.001**	0.15	**0.005**	0.17	**<0.001**
Length	0.08	**<0.001**	0.03	0.096	0.20	**<0.001**	-0.01	0.553	0.00	0.928	-0.02	0.490
Emotionality	-0.01	0.437	0.01	0.748	-0.01	0.466	-0.01	0.686	0.00	0.969	0.00	0.838
Valence	0.04	**<0.001**	0.05	**0.004**	0.04	**0.007**	-0.00	0.842	0.01	0.699	0.01	0.624
Arousal	0.02	**0.035**	-0.01	0.489	0.03	**0.030**	0.05	**0.003**	0.05	0.064	0.05	**0.009**
Concreteness	0.09	**<0.001**	0.01	0.518	0.11	**<0.001**	0.08	**<0.001**	0.11	**<0.001**	0.07	**0.002**
Log Polysemy	0.02	0.091	-0.01	0.671	0.01	0.592	-0.19	**<0.001**	-0.09	**0.016**	-0.22	**<0.001**
**Age of Acquisition (AoA)**	-0.22	**<0.001**	-0.14	**<0.001**	-0.24	**<0.001**	-0.25	**<0.001**	-0.16	**<0.001**	-0.32	**<0.001**
**Semantic Processing (RT)**			-0.09	**<0.001**					-0.07	**0.005**		
**Lexical Decision (RT)**					-0.05	**0.012**					0.04	0.054
Observations	8133		2786		4847		2845		887		2046	
R^2^/R^2^	0.248/0.247		0.279/0.276		0.255/0.253		0.447/0.445		0.296/0.288		0.477/0.475	

Due to constraints of data size, the starting year for the Google Ngram Corpus is 1800 while the starting year for the COHA is 1820. All variables are scaled and mean centered.

**Fig. 3:**
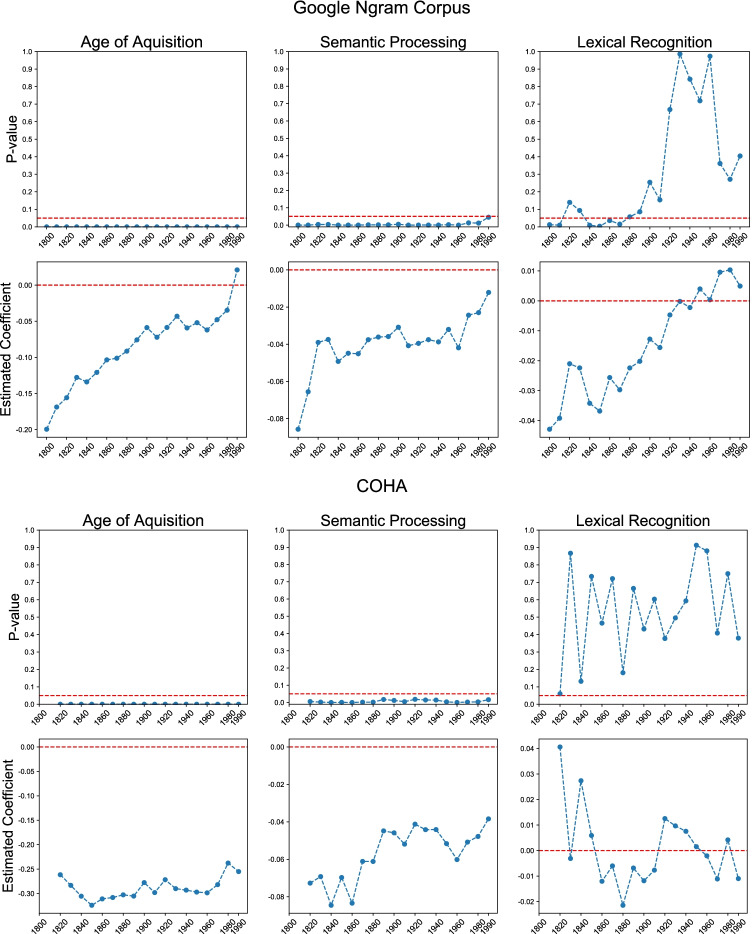
Sensitivity analysis for models that predicted rate of semantic change as inferred from the Google Ngram Corpus (top panel) and the COHA (bottom panel). The *x*-axis represents the historical year of comparison when computing semantic stability. The *y*-axes are *p* values and regression coefficients from the regression analyses. The red dotted lines represent significance threshold of 0.05 for the *p* values and 0 for regression coefficient. (Color figure online)

Semantic similarity was computed using the cosine similarity between word embeddings of 1800^5^ and 2000. We chose year 1800 to be the historical point at which the contemporary meaning was compared against in order to provide sufficient information with respect to the historical dynamics of words. More importantly, it also means that AoA and performance in semantic decision task and lexical decision task should be less likely influenced by the rate of semantic change since 1800 because how people today learn and process language should not be directly influenced by a semantic history that they have never experienced. In addition, we conducted a sensitivity analysis to test if our result is robust to the choice of the historical reference point (see Fig. 3).

Age of acquisition was included in the models that explained the variance of response time of lexical recognition and semantic processing because it has been previously found to be a strong predictor (Morrison & Ellis, 1995). Since we quantified semantic change between year 1800 and 2000, we also included frequency of words at both the start and end years of semantic change in the regression analysis. Frequency was retrieved from the Google Ngram Book Corpus (Michel et al., 2011). Length of word was computed by counting the number of letters in a word. Emotionality was computed by taking the absolute value of the difference between the word’s valence and average valence in the dataset so that the most negative and the most positive words have the largest scores on emotionality. Valence, arousal, and concreteness norms were obtained from Hollis et al. (2017).

Lastly, considering that previous research has found that polysemous words experience higher rates of semantic change, we included polysemy as a covariate in our regression model. Following Hamilton et al. (2016), we quantified polysemy of a word as the clustering coefficient of its network space. We constructed an empirical co-occurrence network for the 50,000 words in the database where words are connected if they co-occur more than one would expect by chance (PPMI > 0; PPMI defined in Equation 1). A word with a larger clustering coefficient means that its semantic neighborhood is more densely connected, and thus it has a low polysemy score. On the other hand, polysemous words have low clustering coefficients since they tend to appear in disjointed or unrelated contexts (i.e., less densely connected semantic neighborhood).

### Results

We first examined multicollinearity for all models. Multicollinearity can be assessed by the variance inflation factor (VIF), which measures how much the variance of a regression coefficient is inflated due to multicollinearity in the model. We computed VIF for each independent variable. The smallest possible VIF value is 1, suggesting complete absence of multicollinearity. As a rule of thumb, a VIF value that exceeds 5 indicates a problematic amount of collinearity (James et al., 2013). We found that all independent variables in all models had a VIF value smaller than 5 (see Appendix Figure 3.4, Fig. 3.5 for correlation tables of all variables).

The regression analysis showed that AoA and semantic processing speed remained strong predictors of semantic stability even with the inclusion of control variables. The results are consistent across semantic stability inferred from different corpora (Table 2). It supports our hypothesis that words acquired later in life experienced greater semantic change (H1.1) and that words whose semantics were processed more slowly also experienced greater semantic change (H1.2). In contrast, reaction speed in the lexical decision task was a significant predictor when the Google Ngram Corpus was used to quantify rate of semantic change, but not a significant predictor when the COHA was used (H1.3).

#### Sensitivity analysis

Before proceeding, it is important to examine whether and how the choice of the year of comparison might alter our results. Given that there is no nonarbitrary way to select the most appropriate year of comparison, we recomputed semantic stability by varying the year of comparison from 1800 to 1990. For each choice of the year of comparison, we performed the same regression analysis as in Table 2 to investigate how the relation between semantic stability and language acquisition (age of acquisition) and between semantic stability and processing (semantic processing and lexical decision) might vary as a function of the historical year selected when computing semantic stability. Overall, the rate of semantic change as quantified from the two corpora showed convergent results (Fig. 3): Age of acquisition and speed of semantic processing are significant predictors of semantic change across all choices of the historical year of comparison. In contrast, we found that the relation between lexical recognition speed and semantic change was highly unstable.

We also performed a sensitivity analysis on how accuracy rates for the semantic decision and lexical decision tasks might vary as a function of the choice of the year of comparison (Appendix Fig. 3.1). The results do not completely align with reaction speed. For the Google Ngram Corpus, higher accuracy in both lexical decision and semantic decision task predicted greater semantic stability. However, for the COHA, higher accuracy in semantic decision task only predicted greater semantic stability when reference year was before 1880. As compared with reaction time, accuracy rate is a less informative cue to the efficiency of lexical and semantic processing because these tasks are very easy to native speakers and consequently variance in accuracy rates is very small (median of accuracy rate is 95% for lexical decision task and 90% for semantic processing task).

## Study 2

In Study 1, we quantified semantic similarity by comparing a word’s meaning in year 1800 to its meaning in year 2000. This allowed us to focus on semantic change that were not directly experienced by people today. However, when semantic change occurs during one’s lifetime, it may make it harder to process the meaning of words because updating words with new meaning could be cognitively costly (Maciejewski et al., 2020).

To answer the question of whether semantic change comes with a cognitive cost, we studied whether semantically unstable words, when compared to semantically stable words, are harder for middle-aged adults to process, but not younger adults. We were unable to do this using the data in Study 1 because the English Lexicon Project and the Calgary Semantic Decision Project recruited participants from an undergraduate student population. Therefore, in Study 2 we use lexical recognition data from the English Crowdsourcing Project (Mandera et al., 2019) that included participants from a wide range of ages (*M* = 35, *SD* = 17). Since there is no existing database that includes the performance of both younger and middle-aged adults on the semantic decision task, we conducted an experiment to obtain this data ourselves. The research was conducted with ethics review board approval from the Max Planck Institute for Human Development. We provide our data online (https://osf.io/gw8vj/).

### Method

**Fig. 4:**
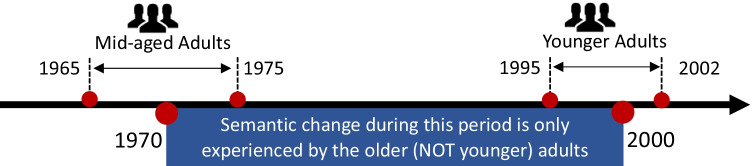
Timeline display of the range of birth year for middle-aged adults and younger adults, and the window during which semantic stability is quantified. (Color figure online)

We computed semantic stability of words between 1970 and 2000 using Equation 2. We recruited middle-aged adults between the ages 45 and 55 (born between 1965 and 1975) and younger adults aged between 18 and 25 (born between 1995 and 2002) so that only middle-aged adults personally experienced the semantic change between 1970 and 2000 (see Fig. 4 for illustration). If semantic change during one's lifetime hampers semantic processing, we should find that semantic change slows down semantic processing speed only for middle-aged adults but not for younger adults (H2.1). The semantic change after year 2000 has been experienced by both younger and older adults, and therefore should not influence the two groups differently.

Following a standard procedure of semantic decision task as described in Pexman et al. (2017), we asked participants to respond, as quickly as possible, whether a word is concrete or abstract. The word stimuli consist of 100 concrete words and 100 abstract words from Brysbaert et al.’s (2014) concreteness ratings for a comprehensive list of English words. For both concrete and abstract groups, 50% of words are semantically stable and 50% are semantically unstable. Semantically stable words were selected from words with stability^1970,2000^ (w) > 0.8, whereas semantically unstable words were selected from words with stability^1970,2000^ (w) < 0.65 (refer to the computation of stability in Equation 2). The words were carefully selected to ensure no significant differences in concreteness, frequency, valence, and age of acquisition between semantically stable words and unstable words, and no significant differences in semantic stability, frequency, valence, and age of acquisition between concrete words and abstract words (see Appendix Table 4.1 for more details). As in Study 1, we included these factors as covariates in the regression models.

We recruited 237 native speakers of English (120 between 18 and 25 years old; 117 between 45 and 55 years old) from Prolific, a crowdsourced data collection platform for psychological research. Each participant responded to each of the 200 words that appeared on the computer screen in random order and decided whether the word was “concrete” or “abstract” by clicking ‘Z’ on the keyboard to indicate abstract or ‘M’ to indicate concrete. The response time was recorded. A randomly selected list of 20 words (10 abstract and 10 concrete) from the stimuli list were used as practice trials for participants to familiarize themselves with the task. We excluded words used in the practice trials from the analysis. The final number of words included in the analysis was 180.

Since Study 2 explores the effect of semantic change throughout the lifetime on word processing and recognition, we regressed semantic stability on semantic processing reaction times and lexical recognition reaction times together with other related variables including log frequency, length, emotionality, arousal, valence, and concreteness. To explore whether middle-aged adults were more sensitive to semantic stability, we also included the participant’s age in the regression model. Unlike Study 1, in which reaction time was aggregated by words (i.e., reaction time of a word was the mean of all participants’ responses), in Study 2, we analyzed the unaggregated, trial-level data using linear mixed effects models. We included both participant and word as random intercept effects. The participant random effect controls for an individual’s idiosyncratic factors underlying responses to all words by the same participant. The word random effect controls for the common factors driving response time from all participants to the same word. In the mixed effect model that predicted lexical recognition, we only included the word random effect and not the participant random effect because the average number of people who responded to the same word was too low (i.e., less than 5 responses per word).

### Results

Using two different datasets from the semantic decision task and lexical decision task , our trial-level regression analysis in Study 2 mirrored our finding in Study 1 (see Table 3, Model 1 and Model 2). Specifically, semantic stability has a significant main effect on semantic processing, such that an increase of one standard deviation of semantic stability leads to a reduction of 20.8 milliseconds in semantic decision RTs (*b* = −20.8), *t*(171) = −3.19, *p* = .002. In contrast, there is no significant main effect of semantic stability on lexical decision performance (*b* = −2.42), *t*(5296) = −1.23, *p* = .218).

**Table 3:** Summary of linear mixed effects models analyzed in Study 2

	**Lexical Recognition RT **		**Semantic Processing RT**			
	*Model 1*		*Model 2*		*Model 3*	
*Predictors*	*Estimates*	*p*	*Estimates*	*p*	*Estimates*	*p*
(Intercept)	915.79	**<0.001**	977.62	**<0.001**	977.61	**<0.001**
Log Frequency (Year 2000)	-63.55	**<0.001**	3.50	0.609	3.71	0.590
Word Length	28.14	**<0.001**	-21.36	**0.002**	-21.36	**0.002**
Age of Acquisition (AOA)	79.17	**<0.001**	10.53	0.105	10.48	0.107
Emotionality	-3.50	0.075	-11.20	0.108	-11.47	0.102
Valence	-6.49	**0.001**	-15.69	**0.018**	-15.83	**0.017**
Arousal	-28.87	**<0.001**	1.27	0.855	1.45	0.835
Concreteness	-7.59	**0.001**	-46.65	**<0.001**	-38.29	**<0.001**
Age (Continuous)	1.86	**<0.001**				
Age (Middle-aged Adults = TRUE)			48.69	**0.043**	48.93	**0.042**
Semantic Stability btw 1970-2000	-2.48	0.218	-20.80	**0.001**	-11.46	0.091
Middle-aged:Semantic Stability					-18.93	**<0.001**
Middle-aged:Concreteness					-16.70	**<0.001**
Concreteness:Semantic Stability					0.38	0.956
Middle-aged:Concreteness:Semantic Stability					-6.59	0.083
**Random Effects**						
σ^2^	327530.75		142385.96		142216.83	
*τ*_00_	17564.11 _Word_		33545.40 _id_		33545.82 _id_	
			6465.37 _Word_		6500.28 _Word_	
ICC	0.05		0.22		0.22	
N	5385 _Word_		237 _id_		237 _id_	
			180 _Word_		180 _Word_	
Observations	3267383		42505		42505	
Marginal R^2^ / Conditional R^2^	0.056/0.104		0.018/0.233		0.019/0.235	

Age is coded as a categorical variable (Younger adults = 0, Middle-aged adults = 1) in the semantic decision task (Models 2 & 3) while it is a continuous variable in lexical decision task (Model 1). All continuous predictors are standardized.

**Fig. 5:**
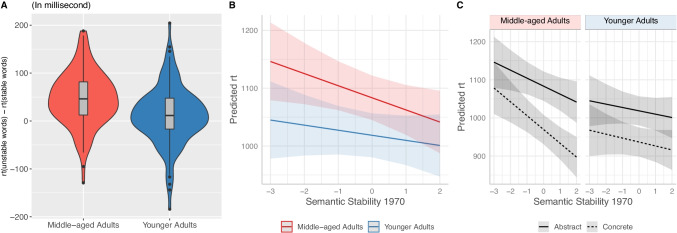
**a** Additional processing time spent on semantically unstable words (compared with semantically stable words). **b** Simple slopes depicting the two-way interaction effect between semantic stability and participant age. **c** Simple slopes depicting the three-way interaction effect between semantic stability, participant age, and concreteness in semantic decision. (Color figure online)

Next, we tested Hypothesis 2.1 regarding whether semantic change slows down processing speed for middle-aged adults but not for younger adults. First, for each participant, we computed the mean of reaction time of semantically stable words and of semantically unstable words. On average, it took middle-aged adults 1,003 milliseconds to process meaning of a semantically stable word, and 46 additional milliseconds (*SD* = 56.8) to process a semantically unstable word. In contrast, younger adults on average took 972 milliseconds to process a semantically stable word, and only 12 additional milliseconds (*SD* = 60.6) to process a semantically unstable word. The additional processing time people spent on processing semantically unstable words (than on semantically stable words) is an indicator of cognitive cost imposed by semantic change (Fig. 5a). Consistent with hypothesis H2.1, we found that this cognitive cost is larger for middle-aged adults than for younger adults, *t*(234) = −4.48, *p* < .001.

Moreover, using a regression model with three-way interaction effect among semantic stability, age group, and concreteness (Table 3, Model 3), we confirmed that the above difference in cognitive cost between middle-aged and younger adults is robust even when other psycholinguistic features are statistically controlled for. We found a significant interaction effect between semantic stability and age (*b* = −18.93), *t*(42,086) = −5.17, *p* < .001. A simple slope analysis (Fig. 5b) shows that middle-aged adults, but not younger adults, responded more slowly to words that were semantically unstable (Middle-aged adults: *b* = −30.7, *SE* = 6.7, *t* = −4.57, *p* < .001; Younger adults: *b* = −11.7, *SE* = 6.7, *t* = −1.74, *p* = .08).

Lastly, we explored whether this semantic-change-imposed processing disadvantage among middle-aged adults was driven by concrete words or abstract words. In a simple slope analysis exploring the three-way interaction between age, concreteness, and semantic stability (Fig. 5c), we found that among middle-aged adults the slope between semantic stability and reaction time was steeper for concrete words (*b* = −38.8, *SE* = 9.4, *t* = −4.14, *p* < .001) than abstract words (*b* = −22.7, *SE* = 9.4, *t* = −2.42, *p* = .02), suggesting that semantic change imposes greater cognitive cost on concrete words than on abstract words.

## Discussion

The present study examined how the semantics of English words evolved under the constraints of human cognition. We first found that cognitive constraints in language acquisition and processing shaped the evolution of a word’s semantics: words acquired later in life or that are more difficult to process in a semantic decision task are under greater selection pressure to change their meanings. Semantic change is an effective strategy for language to meet the everchanging communicative need to express new meanings. However, it is likely to come with a cognitive cost because semantic change is the process of reestablishing the association between meaning and lexical form in the mind of language speakers. We tested this idea in Study 2 and found supporting evidence for it: Semantic change of words, if and only if the change has occurred during one’s lifetime, makes it harder to process the meaning of words.

Using contemporary data on how people process and learn words, our analysis in Study 1 found an association between semantic change and cognitive constraints. It is important to note that since semantic stability in Study 1 was quantified by comparing the contexts of words between year 1800 and year 2000, the historical semantic change of words is largely obscure to most participants who took part in the data collection as they were typically young, college-aged adults recruited from psychology research subject pools. In other words, participants should only be familiar with the modern meanings of words, and it is unlikely for one’s learning history and language performance to be directly influenced by a semantic history that they do not have easy access to. This line of reasoning precludes the conclusion that long-term, historical changes in the meaning of words have strong, direct, measurable effects on the learnability and processing of words. We suggest that it is more plausible to conclude that it is the cognitive constraints associated with learning and using words (as approximated by modern day behavioral measures of age of acquisition and processing performance) that largely shaped the extent of semantic change among words. Specifically, words that are acquired later in life and are more difficult to process in the semantic decision task went through greater semantic change over history.

In Study 1, we showed how cognitive constraints in word learning and processing make some words more likely to change their meanings than others. This result introduced a related question: whether semantic change comes with a cognitive cost—that is, an increased difficulty in processing word meanings because one has to update the mapping between lexical form and meaning. To answer this question, we deliberately designed Study 2 to tease apart the influence of historical semantic change and semantic change within one’s lifespan. This was done by yoking lifespan semantic change to year 1970 so that semantic change could only be directly experienced by middle-aged adults but not younger adults in our study. The results of Study 2 indicated that people were indeed sensitive to changes in a word’s semantics that occurred within their lifetimes. Middle-aged adults, but not younger adults, were slower at processing words that changed their semantics during their lifetime. This suggests that higher rates of semantic change can be costly to cognitive processing. This may be because updating words with new meanings can be cognitively costly (Maciejewski et al., 2020), or alternatively, due to interference effects in memory when multiple, potentially competing, meanings become activated for a single lexical form (Qiu & Johns, 2020; Ramscar et al., 2017).

Language evolution tends to take the path of least resistance (Zipf, 2016); within the present context of semantic change, words tend to change their semantics in a manner that minimizes cognitive effort involved in associating the existing word with a new meaning. Ramiro et al. (2018) provided supporting evidence to this claim by modeling the temporal order in which new senses of individual words emerged over time. Given the set of meanings a word has developed over its semantic history, the authors found that the new meaning that was more likely to emerge next tended to be the meaning with the highest semantic similarity with the existing word sense. They argue that such an evolution path is the most cognitively efficient path because it minimizes the cognitive effort required to associate new meanings with the word.

Our study complements Ramiro et al.’s findings by answering a related question: Which words are more likely to be used to express new meanings than others? The fact that semantic change is more likely to occur among words learned later in life and processed slower may reflect minimization of cognitive effort when new meanings are incorporated into the lexicon. As mentioned in the Introduction, highly semantically stable words are less suitable candidates for hosting new meanings since the cost to reassociate such lexical forms with new meanings would be relatively high. On the other hand, it is less costly to update the meanings of words that are relatively more difficult to learn and process as they are likely to have less well-established form–meaning associations. Hence, one potential way of reducing the overall cost of restructuring and updating the lexicon could be to preferentially assign new meanings to lexical forms that are more difficult to learn and process. Although assigning new meanings to words that are difficult to learn and process could increase the cognitive effort required to then learn and process these words in the short-term (as the results of Study 2 indicate), we emphasize that language evolution involves trade-offs and suggest that this approach ultimately leads to greater long-term benefits such as maintaining the semantics of the core lexicon while enabling languages to be flexible enough to adapt to the emergence of new meanings.

In the present study, we focused our analysis on words that remained in use from 1800 to 2000; otherwise, the computation of semantic stability between 1800 and 2000 would not be possible. That necessarily implies that words that “died” during this period were excluded from the analysis. We speculate that many of these dead words were difficult to learn and process and did not manage to reassociate themselves with new meanings that could have allowed them to survive into the present day. One potential follow-up is to investigate how these dead words differ from the remaining words in terms of learnability, which can be approximated using modern day estimates of the concreteness of words (following Hollis et al., 2017).

As a final point, it is worth highlighting that quantitative research on semantic change in language evolution is usually done with the goal of identifying laws and patterns in historical corpora (Hamilton et al., 2016; Xu & Kemp, 2015). Our approach is different as we aimed to highlight how semantic change could be understood from the perspective of the role of human cognition in language usage, by connecting quantitative patterns of diachronic semantic change to large-scale databases of behavioral measures related to the processing and learning of language. Overall, the present paper provides evidence that semantic evolution of words is related to how early in life the word is acquired and its ease of processing in a semantic decision task. Our results highlight the importance of investigating language evolution with close consideration of the cognitive capabilities and constraints of language users.

## Supplementary Information

ESM 1 (DOCX 4182 kb)

## Footnotes

1 Although it is true that people living today may know that a word had a different meaning hundreds of years ago by reading books written in the past, we argue that obsolete meanings are not easily accessible to people today. We tested this assumption using data from a free-association task (De Deyne et al., 2019) where participants were required to generate three words that first came to their mind when they were presented with a target word. These cue–response patterns represented the mental structure of concepts. By investigating the free associates of a list of selected words that have drastically changed their meaning during the 19th century (e.g*., broadcast, car, awesome*; see complete list of selected words in Appendix Table 1.1 and visualization of their semantic shift in Appendix Fig. 1.1*)*, we did not find associations that directly referred to their obsolete meanings (see Appendix Table 1.2 for lists of associates for each selected word).

2 We present a correlation table of semantic stability as quantified using various methods (SVD and word2vec) and corpora (Google Ngram Corpus and COHA) in Appendix Table 2.2 for readers’ reference.

3 *Broadcast* was shown from 1840 because its frequency was too low before 1840 to train a stable semantic representation.

4 In Kuperman et al. (2012), participants were told to use the following definition when providing AoA ratings for when a given word was learned— “we mean the age at which you would have understood that word if somebody had used it in front of you, EVEN IF YOU DID NOT use, read, or write it at the time.”

5 For rate of semantic change trained on COHA data, the historical point was 1820. This is because COHA data started at 1810 and its data size at 1810 was too small to train a stable model.
