# Supplementary material for: Diachronic semantic change in language is constrained by how people use and learn language
Source: Mem Cognit. 2022 Jun 29;50(6):1284–98. doi: 10.3758/s13421-022-01331-0 (PMC9365724; doi:10.3758/s13421-022-01331-0)
Supplement: Supplementary file 1 — (DOCX 4182 kb) [file 13421_2022_1331_MOESM1_ESM.docx]

Appendix

[Section 1. Testing the assumption that obsolete meanings are not easily accessible to people living today 2](#_Toc93337862)

[Section 2. Justification of our choices on embedding algorithms and corpora 4](#_Toc93337863)

[Section 3. Supplementary materials to Study 1 6](#_Toc93337864)

[Section 4. Supplementary materials to Study 2 10](#_Toc93337865)

# Section 1. Testing the assumption that obsolete meanings are not easily accessible to people living today

Table 1.1: Words that changed their meanings between 1800 and 2000. We consulted the Oxford English Dictionary and <https://www.etymonline.com/> for a rough estimation of the decade when the semantic shift started.


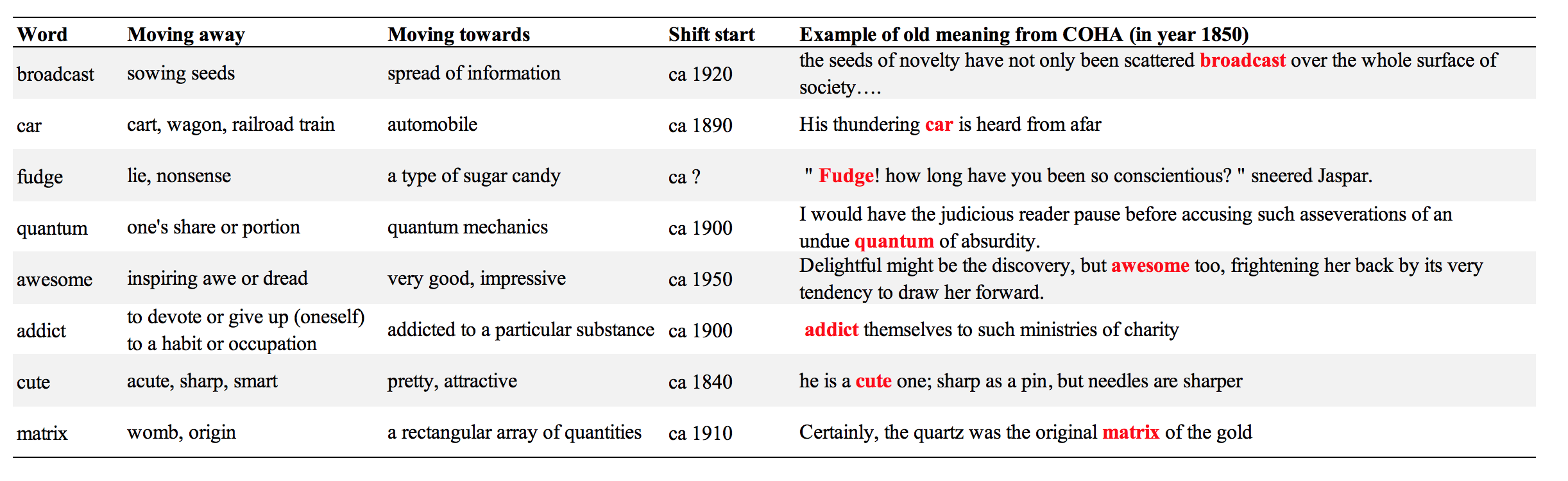

Figure 1.1 Semantic stability of words selected in Appendix Table 1.1. The figure was produced from word embeddings trained by Li et al. (2019) on the Google Ngram Corpus. The black line represents the average semantic similarity across all words in the Macroscope database (Li et al., 2019).

Table 1.2: Free association responses to words in Table 1.1.
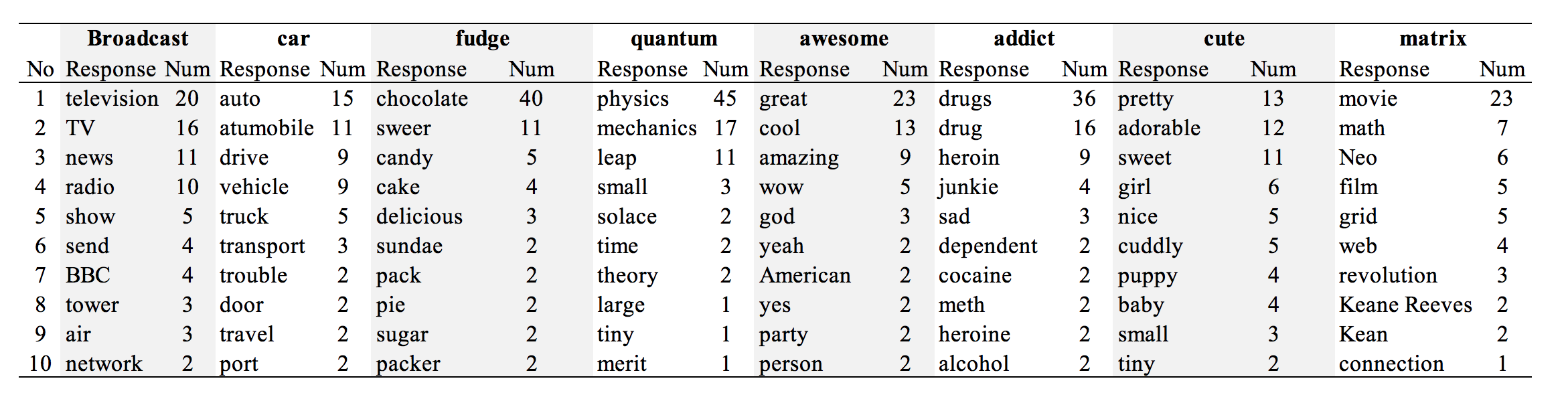


Note: In the free association task (De Deyne et al., 2019), participants were required to generate three words that first came to their mind when they were presented with the cue word. Only the first responses were presented.

# Section 2. Justification of our choices on embedding algorithms and corpora

Table 2.1: Performance of different methods on the detection task (retrieved from Hamilton et al., 2016).


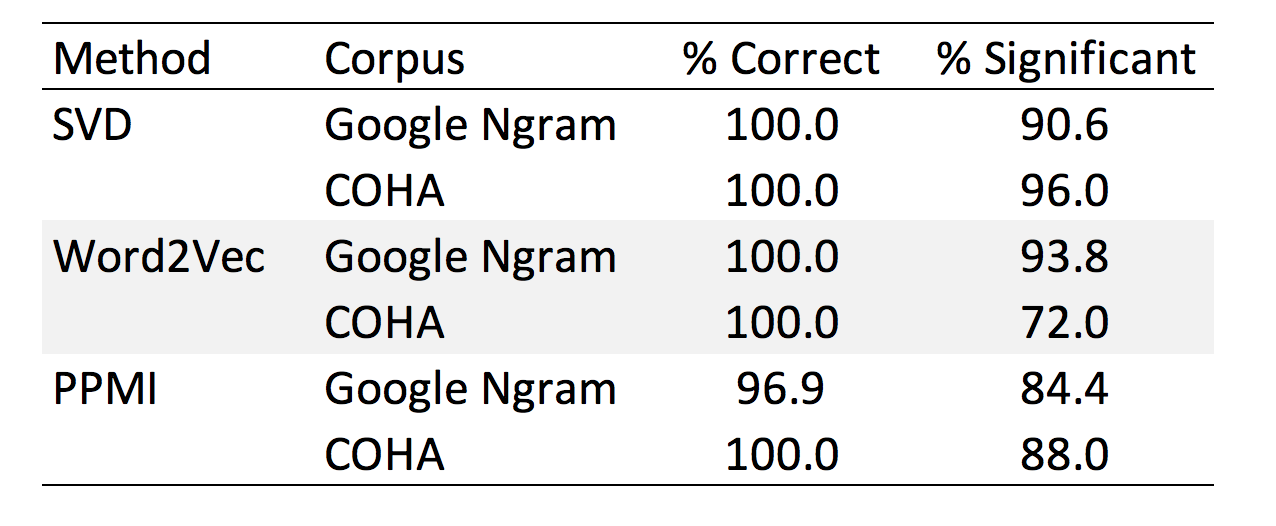


Note: Hamilton et al. (2016) used a set of independently attested semantic shifts as an evaluation set. They evaluated the three methods on both Google Ngram Corpus and COHA. The table above reports the proportion of the correct sign of semantic shifts (e.g., semantic similarity between *gay* and *homosexual* becoming larger instead of smaller), and whether these shifts are statistically significant at the *p < .05* level.

Table 2.2: Correlation table of semantic stability quantified using various algorithms (SVD and word2vec) and corpora (Google Ngram Corpus and COHA).


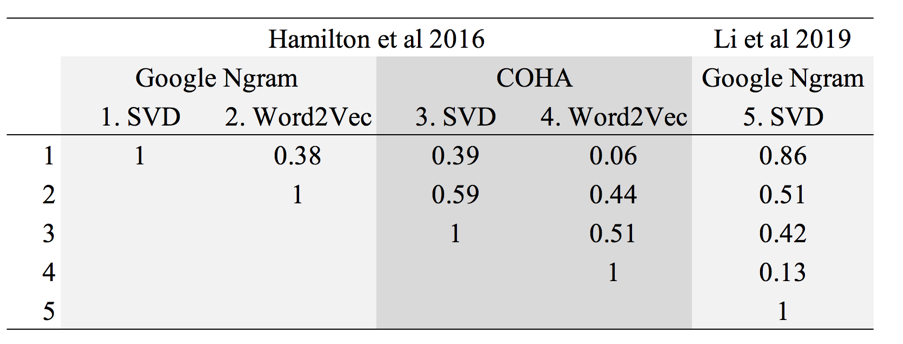


Table 2.3: Correlation between change of frequency between 1850 and 2000 with semantic stability inferred using different corpora and algorithms.


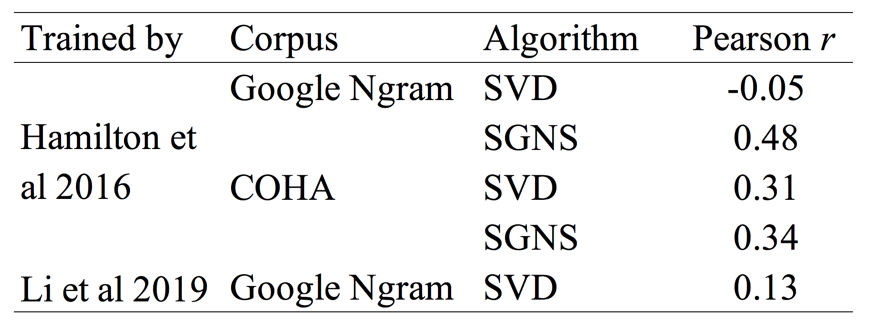


# Section 3. Supplementary materials to Study 1

Figure 3.1: Sensitivity analysis on predicting accuracy of semantic decision task and lexical recognition task in Study 1.

Figure 3.2. Scatter plot of semantic stability between 1800 to 2000 with age of acquisition, response time in semantic decision task, response time in lexical decision task for each word. Linear regression lines are plotted with 95% confidence interval.

Figure 3.3. Marginal effects of age of acquisition, response time in semantic decision task, and response time in lexical decision task on semantic stability of words between 1800 and 2000.

Figure 3.4. Correlation table of variables in the regression model (main text, Table 2, Google Ngram Corpus). Correlations with p-value less than 0.05 are not displayed.

Figure 3.5. Correlation table of variables in the regression model (main text, Table 2, COHA). Correlations with p-value less than 0.05 are not displayed.

# Section 4. Supplementary materials to Study 2

Table 4.1. Results of *t*-tests (*p*-values shown) comparing linguistic features between concrete words and abstract words and between semantically stable words and unstable words in Study 2.

|  | Semantic stability | Concreteness | Log frequency | Valence | AoA | Arousal | Length |
| --- | --- | --- | --- | --- | --- | --- | --- |
| Concrete group vs  Abstract group | 0.87 | **<0.001** | 0.48 | 0.95 | 0.47 | **0.01** | **<0.001** |
| Semantically stable group  vs instable group | **<0.001** | 0.93 | 0.13 | 0.16 | 0.49 | **0.001** | 0.57 |
